# Bis[(4-chloro­benz­yl)triphenyl­phospho­nium] tetra­chloridozincate(II) trihydrate

**DOI:** 10.1107/S1600536810018325

**Published:** 2010-05-22

**Authors:** Kong Wai Tan, Mohd Jamil Maah, Seik Weng Ng

**Affiliations:** a Department of Chemistry, University of Malaya, 50603 Kuala Lumpur, Malaysia

## Abstract

The crystal structure of the title compound, (C_25_H_21_ClP)_2_[ZnCl_4_]·3H_2_O, consists of tetra­hedral phosphonium cations and tetra­hedral zincate anions; the water mol­ecules form weak hydrogen bonds to the anions. Two of the water mol­ecules are disordered over three sites in a 0.68:0.55:0.77 ratio.

## Related literature

For background to phospho­nium tetrahalogenidozincates, see: Bruni *et al.* (1992 ▶). For the crystal structures of two related zincates, see: Aliev *et al.* (1988 ▶); Pattacini *et al.* (2009 ▶).
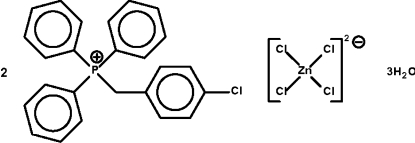


## Experimental

### 

#### Crystal data

(C_25_H_21_ClP)_2_[ZnCl_4_]·3H_2_O
*M* *_r_* = 1036.89
Triclinic, 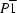

*a* = 11.2634 (12) Å
*b* = 14.2995 (15) Å
*c* = 16.9288 (17) Å
α = 73.651 (1)°
β = 73.527 (2)°
γ = 68.205 (2)°
*V* = 2379.7 (4) Å^3^
*Z* = 2
Mo *K*α radiation
μ = 0.96 mm^−1^
*T* = 100 K
0.15 × 0.10 × 0.03 mm

#### Data collection

Bruker SMART APEX diffractometer
Absorption correction: multi-scan (*SADABS*; Sheldrick, 1996 ▶) *T*_min_ = 0.869, *T*_max_ = 0.972
23163 measured reflections
10895 independent reflections
6579 reflections with *I* > 2σ(*I*)
*R*_int_ = 0.064

#### Refinement

*R*[*F* ^2^ > 2σ(*F* ^2^)] = 0.059
*wR*(*F* ^2^) = 0.164
*S* = 1.00
10895 reflections
568 parameters
18 restraints
H-atom parameters constrained
Δρ_max_ = 1.16 e Å^−3^
Δρ_min_ = −0.82 e Å^−3^

### 

Data collection: *APEX2* (Bruker, 2009 ▶); cell refinement: *SAINT* (Bruker, 2009 ▶); data reduction: *SAINT*; program(s) used to solve structure: *SHELXS97* (Sheldrick, 2008 ▶); program(s) used to refine structure: *SHELXL97* (Sheldrick, 2008 ▶); molecular graphics: *X-SEED* (Barbour, 2001 ▶); software used to prepare material for publication: *publCIF* (Westrip, 2010 ▶).

## Supplementary Material

Crystal structure: contains datablocks global, I. DOI: 10.1107/S1600536810018325/bt5270sup1.cif

Structure factors: contains datablocks I. DOI: 10.1107/S1600536810018325/bt5270Isup2.hkl

Additional supplementary materials:  crystallographic information; 3D view; checkCIF report

## Figures and Tables

**Table 1:** Selected bond lengths (Å)

Zn1—Cl1	2.3049 (14)
Zn1—Cl2	2.2685 (14)
Zn1—Cl3	2.2800 (13)
Zn1—Cl4	2.2571 (12)

**Table 2:** Hydrogen-bond geometry (Å, °)

*D*—H⋯*A*	*D*—H	H⋯*A*	*D*⋯*A*	*D*—H⋯*A*
O1*w*—H1*w*1⋯Cl1	0.84	2.59	3.361 (4)	154
O1*w*—H1*w*2⋯Cl2	0.84	2.41	3.184 (4)	153
O2*w*—H2*w*1⋯Cl1	0.84	2.58	3.385 (9)	161
O3*w*—H3*w*1⋯O4*w*	0.84	2.31	3.12 (2)	160
O3*w*—H3*w*2⋯Cl3	0.84	2.43	3.27 (2)	172
O4*w*—H4*w*1⋯Cl1	0.84	2.29	3.09 (1)	158

**Fig. 1.**
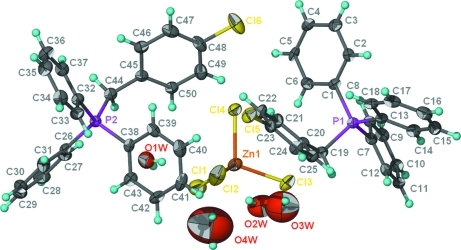
Anisotropic displacement ellipsoid plot (Barbour, 2001) of (C25 H21ClP)2(ZnCl4).3H2O; ellipsoids are drawn at the 50% probability level and H atoms are of arbitrary radius. The disorder in the water molecules is not shown.

## supplementary crystallographic information

### Comment

From far infrared spectral measurements, the anion of the salt, bis[benzyltriphenylphosphonium] tetrachloridozincate, was assigned an ion-pair structure with the cation and anion existing in tetrahedral geometries (Bruni *et al.*, 1992). A earlier synthesis yielded instead the hexachlorodizincate salt, whose formulation was confirmed by crystal structure analysis (Aliev *et al.*, 1988). With the (4-chlorobenzyl)triphenylphosphonium cation, the salt is a tetrachlorozincate; however, the salt this crystallizes as a trihydrate (Scheme I, Fig. 1). The crystal structure consists of tetrahedral cations and tetrahedral anions, with the lattice water molecules being only weakly connected to the anions.

### Experimental

Zinc chloride (0.14 g, 1 mmol) and (4-chlorobenzyl)triphenylphosphonium chloride (0.84 g, 2 mmol) were heated in an ethanol and water mixture ( 3:1, 20 ml) for 3 hours. The yellow compound that separated upon slow evaporation of the solution was recrystallized from a mixture of ethanol and DMF.

### Refinement

Carbon-bound H-atoms were placed in calculated positions (C—H 0.95–0.99 Å) and were included in the refinement in the riding model approximation, with *U*(H) set to 1.2*U*(C).

Tow of the three water molecules are disordered over three positions (O2*w*, O3*w*, O4*w*). As their occupancies refined to nearly 0.68:0.55:0.77, the occupancies were fixed at these values. The anisotropic temperature factors of all water molecules were restrained to be nearly isotropic. Hydrogen atoms were placed in chemically-sensible positions on the basis of possible O–H···Cl interactions; these are weak. For O2*w*, one of its hydrogen atoms occupies the same site as O3*w*, and for O3*w*, one of its hydrogen atoms occupes the same site as O2*w*.

The final difference Fourier map had only one somewhat large peak near O4*w* but was otherwise featureless.

### Crystal data

**Table:**

(C_25_H_21_ClP)_2_[ZnCl_4_]·3H_2_O	*Z* = 2
*M**_r_* = 1036.89	*F*(000) = 1068
Triclinic, *P*1	*D*_x_ = 1.447 Mg m^−^^3^
Hall symbol: -P 1	Mo *K*α radiation, λ = 0.71073 Å
*a* = 11.2634 (12) Å	Cell parameters from 2585 reflections
*b* = 14.2995 (15) Å	θ = 2.5–24.6°
*c* = 16.9288 (17) Å	µ = 0.96 mm^−^^1^
α = 73.651 (1)°	*T* = 100 K
β = 73.527 (2)°	Prism, yellow
γ = 68.205 (2)°	0.15 × 0.10 × 0.03 mm
*V* = 2379.7 (4) Å^3^	

### Data collection

**Table:**

Bruker SMART APEX diffractometer	10895 independent reflections
Radiation source: fine-focus sealed tube	6579 reflections with *I* > 2σ(*I*)
graphite	*R*_int_ = 0.064
ω scans	θ_max_ = 27.5°, θ_min_ = 1.3°
Absorption correction: multi-scan (*SADABS*; Sheldrick, 1996)	*h* = −14→14
*T*_min_ = 0.869, *T*_max_ = 0.972	*k* = −17→18
23163 measured reflections	*l* = −21→19

### Refinement

**Table:**

Refinement on *F*^2^	Primary atom site location: structure-invariant direct methods
Least-squares matrix: full	Secondary atom site location: difference Fourier map
*R*[*F*^2^ > 2σ(*F*^2^)] = 0.059	Hydrogen site location: inferred from neighbouring sites
*wR*(*F*^2^) = 0.164	H-atom parameters constrained
*S* = 1.00	*w* = 1/[σ^2^(*F*_o_^2^) + (0.0725*P*)^2^ + 0.642*P*] where *P* = (*F*_o_^2^ + 2*F*_c_^2^)/3
10895 reflections	(Δ/σ)_max_ = 0.001
568 parameters	Δρ_max_ = 1.16 e Å^−^^3^
18 restraints	Δρ_min_ = −0.82 e Å^−^^3^

### Fractional atomic coordinates and isotropic or equivalent isotropic displacement parameters (Å^2^)

**Table:**

	*x*	*y*	*z*	*U*_iso_*/*U*_eq_	Occ. (<1)
Zn1	0.39614 (5)	0.84375 (4)	0.28532 (3)	0.02513 (15)	
Cl1	0.21085 (13)	0.86435 (11)	0.38977 (8)	0.0408 (3)	
Cl2	0.40060 (15)	0.72339 (10)	0.22031 (8)	0.0422 (3)	
Cl3	0.40802 (12)	0.99004 (9)	0.18953 (7)	0.0305 (3)	
Cl4	0.57229 (10)	0.78183 (9)	0.34554 (7)	0.0256 (3)	
Cl5	0.84826 (11)	0.43139 (8)	−0.02489 (8)	0.0289 (3)	
Cl6	1.00321 (12)	0.47193 (12)	0.24586 (9)	0.0439 (4)	
P1	0.80632 (10)	0.91395 (8)	0.01410 (7)	0.0164 (2)	
P2	0.44814 (10)	0.31622 (9)	0.40672 (7)	0.0172 (2)	
O1w	0.3019 (3)	0.6091 (3)	0.4044 (2)	0.0357 (8)	
H1w1	0.2679	0.6668	0.4182	0.054*	
H1w2	0.3383	0.6183	0.3534	0.054*	
O2w	0.1837 (9)	1.1168 (7)	0.3434 (6)	0.071 (2)	0.68
H2w1	0.2038	1.0540	0.3437	0.106*	0.68
H2w2	0.2266	1.1437	0.2997	0.106*	0.68
O3w	0.2158 (17)	1.1580 (12)	0.3051 (10)	0.131 (6)	0.55
H3w1	0.1791	1.1222	0.3459	0.197*	0.55
H3w2	0.2676	1.1196	0.2720	0.197*	0.55
O4w	0.0156 (13)	1.0658 (10)	0.4421 (8)	0.215 (6)	0.77
H4w1	0.0739	1.0225	0.4154	0.322*	0.77
H4w2	−0.0462	1.0955	0.4162	0.322*	0.77
C1	0.8966 (4)	0.8405 (3)	0.0951 (3)	0.0165 (9)	
C2	1.0319 (4)	0.8094 (3)	0.0784 (3)	0.0181 (9)	
H2	1.0791	0.8212	0.0223	0.022*	
C3	1.0979 (4)	0.7607 (3)	0.1443 (3)	0.0218 (10)	
H3	1.1905	0.7397	0.1332	0.026*	
C4	1.0296 (4)	0.7425 (3)	0.2259 (3)	0.0221 (10)	
H4	1.0755	0.7086	0.2705	0.027*	
C5	0.8946 (4)	0.7736 (3)	0.2428 (3)	0.0212 (10)	
H5	0.8481	0.7610	0.2990	0.025*	
C6	0.8273 (4)	0.8231 (3)	0.1778 (3)	0.0202 (9)	
H6	0.7346	0.8451	0.1894	0.024*	
C7	0.7331 (4)	1.0437 (3)	0.0293 (3)	0.0177 (9)	
C8	0.7498 (4)	1.0733 (3)	0.0960 (3)	0.0187 (9)	
H8	0.8053	1.0250	0.1322	0.022*	
C9	0.6865 (4)	1.1723 (3)	0.1099 (3)	0.0212 (10)	
H9	0.6978	1.1919	0.1558	0.025*	
C10	0.6062 (4)	1.2427 (3)	0.0563 (3)	0.0224 (10)	
H10	0.5619	1.3107	0.0659	0.027*	
C11	0.5903 (4)	1.2146 (3)	−0.0107 (3)	0.0219 (10)	
H11	0.5364	1.2637	−0.0476	0.026*	
C12	0.6518 (4)	1.1160 (3)	−0.0243 (3)	0.0188 (9)	
H12	0.6394	1.0968	−0.0700	0.023*	
C13	0.9112 (4)	0.9036 (3)	−0.0868 (3)	0.0162 (9)	
C14	0.9176 (4)	0.9902 (4)	−0.1498 (3)	0.0226 (10)	
H14	0.8681	1.0569	−0.1387	0.027*	
C15	0.9958 (4)	0.9791 (4)	−0.2285 (3)	0.0252 (10)	
H15	1.0006	1.0383	−0.2709	0.030*	
C16	1.0669 (4)	0.8823 (4)	−0.2454 (3)	0.0270 (11)	
H16	1.1206	0.8747	−0.2993	0.032*	
C17	1.0593 (4)	0.7961 (4)	−0.1833 (3)	0.0235 (10)	
H17	1.1071	0.7295	−0.1950	0.028*	
C18	0.9830 (4)	0.8068 (3)	−0.1046 (3)	0.0202 (9)	
H18	0.9795	0.7474	−0.0623	0.024*	
C19	0.6724 (4)	0.8668 (3)	0.0237 (3)	0.0212 (10)	
H19A	0.6155	0.8714	0.0800	0.025*	
H19B	0.6195	0.9116	−0.0187	0.025*	
C20	0.7178 (4)	0.7573 (3)	0.0118 (3)	0.0190 (9)	
C21	0.7489 (4)	0.6754 (3)	0.0778 (3)	0.0238 (10)	
H21	0.7413	0.6883	0.1314	0.029*	
C22	0.7906 (5)	0.5755 (4)	0.0667 (3)	0.0273 (11)	
H22	0.8135	0.5196	0.1117	0.033*	
C23	0.7986 (4)	0.5579 (3)	−0.0108 (3)	0.0221 (10)	
C24	0.7664 (4)	0.6377 (3)	−0.0771 (3)	0.0211 (10)	
H24	0.7714	0.6245	−0.1301	0.025*	
C25	0.7269 (4)	0.7367 (3)	−0.0653 (3)	0.0211 (10)	
H25	0.7054	0.7922	−0.1109	0.025*	
C26	0.2848 (4)	0.3235 (3)	0.4640 (3)	0.0187 (9)	
C27	0.2169 (4)	0.3938 (4)	0.5163 (3)	0.0240 (10)	
H27	0.2575	0.4372	0.5242	0.029*	
C28	0.0892 (4)	0.4003 (4)	0.5570 (3)	0.0291 (11)	
H28	0.0419	0.4485	0.5926	0.035*	
C29	0.0302 (4)	0.3359 (4)	0.5457 (3)	0.0305 (12)	
H29	−0.0571	0.3402	0.5738	0.037*	
C30	0.0983 (4)	0.2664 (4)	0.4939 (3)	0.0306 (11)	
H30	0.0580	0.2228	0.4861	0.037*	
C31	0.2262 (4)	0.2597 (4)	0.4527 (3)	0.0270 (11)	
H31	0.2733	0.2115	0.4170	0.032*	
C32	0.5400 (4)	0.1858 (3)	0.3999 (3)	0.0186 (9)	
C33	0.5334 (4)	0.1442 (3)	0.3363 (3)	0.0225 (10)	
H33	0.4872	0.1874	0.2935	0.027*	
C34	0.5935 (4)	0.0411 (4)	0.3354 (3)	0.0260 (10)	
H34	0.5867	0.0130	0.2928	0.031*	
C35	0.6635 (4)	−0.0215 (4)	0.3963 (3)	0.0266 (11)	
H35	0.7057	−0.0924	0.3952	0.032*	
C36	0.6728 (5)	0.0184 (4)	0.4591 (3)	0.0283 (11)	
H36	0.7222	−0.0249	0.5003	0.034*	
C37	0.6099 (4)	0.1214 (3)	0.4618 (3)	0.0238 (10)	
H37	0.6143	0.1483	0.5058	0.029*	
C38	0.4406 (4)	0.3890 (3)	0.3018 (3)	0.0172 (9)	
C39	0.5555 (4)	0.3843 (3)	0.2417 (3)	0.0226 (10)	
H39	0.6372	0.3421	0.2560	0.027*	
C40	0.5502 (5)	0.4410 (4)	0.1614 (3)	0.0279 (11)	
H40	0.6286	0.4381	0.1205	0.034*	
C41	0.4312 (5)	0.5023 (3)	0.1397 (3)	0.0269 (11)	
H41	0.4280	0.5411	0.0842	0.032*	
C42	0.3169 (5)	0.5067 (3)	0.1995 (3)	0.0248 (10)	
H42	0.2354	0.5484	0.1847	0.030*	
C43	0.3207 (4)	0.4506 (3)	0.2806 (3)	0.0221 (10)	
H43	0.2422	0.4541	0.3214	0.026*	
C44	0.5220 (4)	0.3699 (3)	0.4574 (3)	0.0201 (9)	
H44A	0.4571	0.4342	0.4732	0.024*	
H44B	0.5432	0.3211	0.5098	0.024*	
C45	0.6443 (4)	0.3932 (3)	0.4048 (3)	0.0180 (9)	
C46	0.7670 (4)	0.3166 (4)	0.3986 (3)	0.0222 (10)	
H46	0.7743	0.2481	0.4281	0.027*	
C47	0.8776 (4)	0.3408 (4)	0.3496 (3)	0.0272 (11)	
H47	0.9608	0.2894	0.3451	0.033*	
C48	0.8645 (4)	0.4404 (4)	0.3075 (3)	0.0255 (11)	
C49	0.7463 (4)	0.5175 (4)	0.3128 (3)	0.0261 (10)	
H49	0.7400	0.5860	0.2839	0.031*	
C50	0.6373 (4)	0.4922 (3)	0.3612 (3)	0.0199 (9)	
H50	0.5548	0.5443	0.3648	0.024*	

### Atomic displacement parameters (Å^2^)

**Table:**

	*U*^11^	*U*^22^	*U*^33^	*U*^12^	*U*^13^	*U*^23^
Zn1	0.0277 (3)	0.0273 (3)	0.0197 (3)	−0.0064 (2)	−0.0081 (2)	−0.0036 (2)
Cl1	0.0356 (7)	0.0486 (9)	0.0346 (8)	−0.0098 (6)	−0.0089 (6)	−0.0055 (6)
Cl2	0.0713 (10)	0.0370 (8)	0.0281 (7)	−0.0240 (7)	−0.0180 (7)	−0.0040 (6)
Cl3	0.0436 (7)	0.0241 (6)	0.0228 (6)	−0.0078 (5)	−0.0130 (5)	−0.0009 (5)
Cl4	0.0240 (5)	0.0269 (6)	0.0229 (6)	−0.0062 (5)	−0.0070 (4)	−0.0002 (5)
Cl5	0.0349 (6)	0.0157 (6)	0.0341 (7)	−0.0043 (5)	−0.0100 (5)	−0.0038 (5)
Cl6	0.0323 (7)	0.0622 (10)	0.0424 (8)	−0.0276 (7)	0.0140 (6)	−0.0226 (7)
P1	0.0163 (5)	0.0170 (6)	0.0162 (6)	−0.0039 (4)	−0.0041 (4)	−0.0048 (5)
P2	0.0170 (5)	0.0177 (6)	0.0163 (6)	−0.0050 (4)	−0.0028 (4)	−0.0038 (5)
O1w	0.0296 (18)	0.035 (2)	0.036 (2)	−0.0071 (15)	0.0008 (15)	−0.0087 (17)
O2w	0.074 (5)	0.066 (5)	0.075 (5)	−0.003 (4)	−0.024 (4)	−0.035 (4)
O3w	0.135 (9)	0.138 (10)	0.131 (10)	−0.038 (7)	−0.033 (7)	−0.040 (7)
O4w	0.199 (9)	0.251 (10)	0.215 (9)	−0.053 (7)	−0.065 (7)	−0.078 (7)
C1	0.021 (2)	0.014 (2)	0.017 (2)	−0.0041 (17)	−0.0069 (17)	−0.0043 (17)
C2	0.020 (2)	0.017 (2)	0.018 (2)	−0.0067 (17)	−0.0010 (17)	−0.0064 (18)
C3	0.023 (2)	0.015 (2)	0.027 (3)	−0.0025 (18)	−0.0071 (19)	−0.0049 (19)
C4	0.026 (2)	0.018 (2)	0.021 (2)	−0.0045 (18)	−0.0101 (19)	−0.0001 (19)
C5	0.031 (2)	0.015 (2)	0.014 (2)	−0.0064 (18)	−0.0043 (18)	0.0016 (18)
C6	0.016 (2)	0.019 (2)	0.023 (2)	−0.0039 (17)	−0.0015 (18)	−0.0068 (19)
C7	0.016 (2)	0.021 (2)	0.016 (2)	−0.0055 (17)	0.0007 (16)	−0.0070 (18)
C8	0.016 (2)	0.022 (2)	0.018 (2)	−0.0061 (17)	−0.0030 (17)	−0.0054 (19)
C9	0.021 (2)	0.026 (2)	0.019 (2)	−0.0072 (19)	−0.0002 (18)	−0.011 (2)
C10	0.021 (2)	0.017 (2)	0.028 (3)	−0.0043 (18)	−0.0030 (19)	−0.007 (2)
C11	0.016 (2)	0.019 (2)	0.026 (3)	−0.0027 (17)	−0.0052 (18)	0.0002 (19)
C12	0.019 (2)	0.023 (2)	0.016 (2)	−0.0092 (18)	0.0001 (17)	−0.0063 (19)
C13	0.018 (2)	0.020 (2)	0.014 (2)	−0.0058 (17)	−0.0063 (17)	−0.0062 (18)
C14	0.021 (2)	0.024 (2)	0.022 (2)	−0.0045 (19)	−0.0044 (18)	−0.007 (2)
C15	0.025 (2)	0.032 (3)	0.019 (2)	−0.012 (2)	−0.0032 (19)	−0.002 (2)
C16	0.020 (2)	0.044 (3)	0.019 (2)	−0.011 (2)	−0.0007 (18)	−0.012 (2)
C17	0.018 (2)	0.031 (3)	0.022 (2)	−0.0011 (19)	−0.0035 (18)	−0.017 (2)
C18	0.019 (2)	0.022 (2)	0.021 (2)	−0.0033 (18)	−0.0081 (18)	−0.0061 (19)
C19	0.020 (2)	0.021 (2)	0.023 (2)	−0.0052 (18)	−0.0077 (18)	−0.0027 (19)
C20	0.016 (2)	0.017 (2)	0.025 (2)	−0.0065 (17)	−0.0047 (18)	−0.0036 (19)
C21	0.031 (2)	0.025 (3)	0.019 (2)	−0.014 (2)	−0.0062 (19)	−0.001 (2)
C22	0.041 (3)	0.022 (3)	0.024 (3)	−0.017 (2)	−0.015 (2)	0.007 (2)
C23	0.023 (2)	0.016 (2)	0.027 (3)	−0.0085 (18)	−0.0034 (19)	−0.0026 (19)
C24	0.026 (2)	0.020 (2)	0.019 (2)	−0.0060 (18)	−0.0047 (18)	−0.0070 (19)
C25	0.023 (2)	0.019 (2)	0.021 (2)	−0.0063 (18)	−0.0066 (18)	−0.0021 (19)
C26	0.018 (2)	0.019 (2)	0.017 (2)	−0.0044 (17)	−0.0057 (17)	0.0007 (18)
C27	0.020 (2)	0.029 (3)	0.023 (3)	−0.0060 (19)	−0.0081 (19)	−0.004 (2)
C28	0.022 (2)	0.037 (3)	0.021 (3)	0.000 (2)	−0.0045 (19)	−0.007 (2)
C29	0.018 (2)	0.049 (3)	0.018 (3)	−0.011 (2)	−0.0052 (19)	0.005 (2)
C30	0.026 (2)	0.036 (3)	0.032 (3)	−0.016 (2)	−0.010 (2)	0.002 (2)
C31	0.031 (3)	0.023 (3)	0.027 (3)	−0.011 (2)	−0.003 (2)	−0.004 (2)
C32	0.020 (2)	0.019 (2)	0.017 (2)	−0.0048 (17)	−0.0042 (17)	−0.0042 (18)
C33	0.025 (2)	0.022 (2)	0.022 (2)	−0.0065 (19)	−0.0083 (19)	−0.004 (2)
C34	0.033 (3)	0.029 (3)	0.020 (3)	−0.012 (2)	−0.003 (2)	−0.009 (2)
C35	0.032 (3)	0.018 (2)	0.028 (3)	−0.005 (2)	−0.006 (2)	−0.006 (2)
C36	0.034 (3)	0.024 (3)	0.025 (3)	−0.004 (2)	−0.011 (2)	−0.002 (2)
C37	0.024 (2)	0.025 (3)	0.023 (3)	−0.0075 (19)	−0.0028 (19)	−0.007 (2)
C38	0.025 (2)	0.017 (2)	0.012 (2)	−0.0090 (18)	−0.0023 (17)	−0.0039 (17)
C39	0.020 (2)	0.027 (3)	0.024 (3)	−0.0115 (19)	−0.0007 (19)	−0.010 (2)
C40	0.036 (3)	0.034 (3)	0.020 (3)	−0.019 (2)	0.001 (2)	−0.009 (2)
C41	0.048 (3)	0.019 (2)	0.018 (2)	−0.016 (2)	−0.009 (2)	−0.0003 (19)
C42	0.032 (2)	0.020 (2)	0.022 (3)	−0.004 (2)	−0.013 (2)	−0.002 (2)
C43	0.025 (2)	0.019 (2)	0.020 (2)	−0.0037 (18)	−0.0031 (19)	−0.0063 (19)
C44	0.022 (2)	0.021 (2)	0.018 (2)	−0.0062 (18)	−0.0043 (18)	−0.0053 (19)
C45	0.023 (2)	0.022 (2)	0.014 (2)	−0.0086 (18)	−0.0075 (17)	−0.0061 (18)
C46	0.024 (2)	0.023 (2)	0.021 (2)	−0.0053 (19)	−0.0082 (19)	−0.006 (2)
C47	0.021 (2)	0.035 (3)	0.029 (3)	−0.007 (2)	−0.005 (2)	−0.016 (2)
C48	0.023 (2)	0.042 (3)	0.020 (2)	−0.018 (2)	0.0044 (19)	−0.016 (2)
C49	0.032 (3)	0.027 (3)	0.023 (3)	−0.016 (2)	−0.002 (2)	−0.007 (2)
C50	0.021 (2)	0.020 (2)	0.019 (2)	−0.0046 (18)	−0.0058 (18)	−0.0054 (19)

### Geometric parameters (Å, °)

**Table:**

Zn1—Cl1	2.3049 (14)	C19—H19B	0.9900
Zn1—Cl2	2.2685 (14)	C20—C25	1.385 (6)
Zn1—Cl3	2.2800 (13)	C20—C21	1.387 (6)
Zn1—Cl4	2.2571 (12)	C21—C22	1.379 (6)
Cl5—C23	1.749 (4)	C21—H21	0.9500
Cl6—C48	1.752 (4)	C22—C23	1.376 (6)
P1—C7	1.790 (4)	C22—H22	0.9500
P1—C13	1.790 (4)	C23—C24	1.377 (6)
P1—C1	1.790 (4)	C24—C25	1.372 (6)
P1—C19	1.820 (4)	C24—H24	0.9500
P2—C32	1.784 (4)	C25—H25	0.9500
P2—C38	1.796 (4)	C26—C31	1.387 (6)
P2—C26	1.799 (4)	C26—C27	1.388 (6)
P2—C44	1.807 (4)	C27—C28	1.389 (6)
O1w—H1w1	0.8401	C27—H27	0.9500
O1w—H1w2	0.8400	C28—C29	1.396 (7)
O2w—H2w1	0.8400	C28—H28	0.9500
O2w—H2w2	0.8400	C29—C30	1.375 (7)
O3w—H3w1	0.8399	C29—H29	0.9500
O3w—H3w2	0.8400	C30—C31	1.391 (6)
O4w—H4w1	0.8399	C30—H30	0.9500
O4w—H4w2	0.8400	C31—H31	0.9500
C1—C2	1.387 (6)	C32—C37	1.396 (6)
C1—C6	1.400 (6)	C32—C33	1.399 (6)
C2—C3	1.390 (6)	C33—C34	1.378 (6)
C2—H2	0.9500	C33—H33	0.9500
C3—C4	1.382 (6)	C34—C35	1.380 (6)
C3—H3	0.9500	C34—H34	0.9500
C4—C5	1.383 (6)	C35—C36	1.382 (6)
C4—H4	0.9500	C35—H35	0.9500
C5—C6	1.387 (6)	C36—C37	1.385 (6)
C5—H5	0.9500	C36—H36	0.9500
C6—H6	0.9500	C37—H37	0.9500
C7—C8	1.391 (6)	C38—C39	1.393 (6)
C7—C12	1.405 (6)	C38—C43	1.394 (6)
C8—C9	1.382 (6)	C39—C40	1.378 (6)
C8—H8	0.9500	C39—H39	0.9500
C9—C10	1.390 (6)	C40—C41	1.387 (7)
C9—H9	0.9500	C40—H40	0.9500
C10—C11	1.381 (6)	C41—C42	1.386 (7)
C10—H10	0.9500	C41—H41	0.9500
C11—C12	1.374 (6)	C42—C43	1.385 (6)
C11—H11	0.9500	C42—H42	0.9500
C12—H12	0.9500	C43—H43	0.9500
C13—C18	1.388 (6)	C44—C45	1.510 (6)
C13—C14	1.398 (6)	C44—H44A	0.9900
C14—C15	1.388 (6)	C44—H44B	0.9900
C14—H14	0.9500	C45—C50	1.385 (6)
C15—C16	1.382 (6)	C45—C46	1.406 (6)
C15—H15	0.9500	C46—C47	1.391 (6)
C16—C17	1.389 (7)	C46—H46	0.9500
C16—H16	0.9500	C47—C48	1.375 (7)
C17—C18	1.379 (6)	C47—H47	0.9500
C17—H17	0.9500	C48—C49	1.376 (6)
C18—H18	0.9500	C49—C50	1.379 (6)
C19—C20	1.509 (6)	C49—H49	0.9500
C19—H19A	0.9900	C50—H50	0.9500
			
Cl4—Zn1—Cl2	108.29 (5)	C23—C22—H22	120.5
Cl4—Zn1—Cl3	108.25 (5)	C21—C22—H22	120.5
Cl2—Zn1—Cl3	110.16 (5)	C22—C23—C24	121.5 (4)
Cl4—Zn1—Cl1	108.61 (5)	C22—C23—Cl5	119.2 (3)
Cl2—Zn1—Cl1	106.56 (6)	C24—C23—Cl5	119.3 (3)
Cl3—Zn1—Cl1	114.79 (5)	C25—C24—C23	118.8 (4)
C7—P1—C13	112.9 (2)	C25—C24—H24	120.6
C7—P1—C1	108.51 (19)	C23—C24—H24	120.6
C13—P1—C1	109.85 (19)	C24—C25—C20	121.3 (4)
C7—P1—C19	106.25 (19)	C24—C25—H25	119.4
C13—P1—C19	109.2 (2)	C20—C25—H25	119.4
C1—P1—C19	110.1 (2)	C31—C26—C27	120.4 (4)
C32—P2—C38	107.93 (19)	C31—C26—P2	118.6 (3)
C32—P2—C26	109.4 (2)	C27—C26—P2	120.9 (3)
C38—P2—C26	108.72 (19)	C26—C27—C28	119.5 (4)
C32—P2—C44	111.9 (2)	C26—C27—H27	120.3
C38—P2—C44	109.3 (2)	C28—C27—H27	120.3
C26—P2—C44	109.7 (2)	C27—C28—C29	120.1 (5)
H1w1—O1w—H1w2	107.7	C27—C28—H28	119.9
H2w1—O2w—H2w2	108.8	C29—C28—H28	119.9
H3w1—O3w—H3w2	108.2	C30—C29—C28	120.0 (4)
H4w1—O4w—H4w2	109.0	C30—C29—H29	120.0
C2—C1—C6	120.2 (4)	C28—C29—H29	120.0
C2—C1—P1	121.3 (3)	C29—C30—C31	120.2 (5)
C6—C1—P1	118.3 (3)	C29—C30—H30	119.9
C1—C2—C3	119.4 (4)	C31—C30—H30	119.9
C1—C2—H2	120.3	C26—C31—C30	119.8 (4)
C3—C2—H2	120.3	C26—C31—H31	120.1
C4—C3—C2	120.5 (4)	C30—C31—H31	120.1
C4—C3—H3	119.8	C37—C32—C33	119.0 (4)
C2—C3—H3	119.8	C37—C32—P2	120.7 (3)
C3—C4—C5	120.2 (4)	C33—C32—P2	120.0 (3)
C3—C4—H4	119.9	C34—C33—C32	120.5 (4)
C5—C4—H4	119.9	C34—C33—H33	119.8
C4—C5—C6	120.0 (4)	C32—C33—H33	119.8
C4—C5—H5	120.0	C33—C34—C35	120.0 (4)
C6—C5—H5	120.0	C33—C34—H34	120.0
C5—C6—C1	119.7 (4)	C35—C34—H34	120.0
C5—C6—H6	120.2	C34—C35—C36	120.4 (4)
C1—C6—H6	120.2	C34—C35—H35	119.8
C8—C7—C12	119.2 (4)	C36—C35—H35	119.8
C8—C7—P1	121.2 (3)	C35—C36—C37	120.0 (4)
C12—C7—P1	119.5 (3)	C35—C36—H36	120.0
C9—C8—C7	120.6 (4)	C37—C36—H36	120.0
C9—C8—H8	119.7	C36—C37—C32	120.1 (4)
C7—C8—H8	119.7	C36—C37—H37	120.0
C8—C9—C10	119.4 (4)	C32—C37—H37	120.0
C8—C9—H9	120.3	C39—C38—C43	120.1 (4)
C10—C9—H9	120.3	C39—C38—P2	119.7 (3)
C11—C10—C9	120.5 (4)	C43—C38—P2	120.3 (3)
C11—C10—H10	119.8	C40—C39—C38	119.8 (4)
C9—C10—H10	119.8	C40—C39—H39	120.1
C12—C11—C10	120.4 (4)	C38—C39—H39	120.1
C12—C11—H11	119.8	C39—C40—C41	120.5 (4)
C10—C11—H11	119.8	C39—C40—H40	119.7
C11—C12—C7	119.9 (4)	C41—C40—H40	119.7
C11—C12—H12	120.0	C42—C41—C40	119.6 (4)
C7—C12—H12	120.0	C42—C41—H41	120.2
C18—C13—C14	119.0 (4)	C40—C41—H41	120.2
C18—C13—P1	119.1 (3)	C43—C42—C41	120.5 (4)
C14—C13—P1	121.8 (3)	C43—C42—H42	119.7
C15—C14—C13	120.3 (4)	C41—C42—H42	119.7
C15—C14—H14	119.9	C42—C43—C38	119.4 (4)
C13—C14—H14	119.9	C42—C43—H43	120.3
C16—C15—C14	120.2 (4)	C38—C43—H43	120.3
C16—C15—H15	119.9	C45—C44—P2	114.8 (3)
C14—C15—H15	119.9	C45—C44—H44A	108.6
C15—C16—C17	119.6 (4)	P2—C44—H44A	108.6
C15—C16—H16	120.2	C45—C44—H44B	108.6
C17—C16—H16	120.2	P2—C44—H44B	108.6
C18—C17—C16	120.4 (4)	H44A—C44—H44B	107.5
C18—C17—H17	119.8	C50—C45—C46	118.4 (4)
C16—C17—H17	119.8	C50—C45—C44	119.9 (4)
C17—C18—C13	120.5 (4)	C46—C45—C44	121.7 (4)
C17—C18—H18	119.7	C47—C46—C45	120.2 (4)
C13—C18—H18	119.7	C47—C46—H46	119.9
C20—C19—P1	113.1 (3)	C45—C46—H46	119.9
C20—C19—H19A	109.0	C48—C47—C46	118.8 (4)
P1—C19—H19A	109.0	C48—C47—H47	120.6
C20—C19—H19B	109.0	C46—C47—H47	120.6
P1—C19—H19B	109.0	C47—C48—C49	122.6 (4)
H19A—C19—H19B	107.8	C47—C48—Cl6	119.2 (4)
C25—C20—C21	118.6 (4)	C49—C48—Cl6	118.2 (4)
C25—C20—C19	120.4 (4)	C48—C49—C50	117.9 (4)
C21—C20—C19	121.0 (4)	C48—C49—H49	121.0
C22—C21—C20	120.8 (4)	C50—C49—H49	121.0
C22—C21—H21	119.6	C49—C50—C45	122.1 (4)
C20—C21—H21	119.6	C49—C50—H50	119.0
C23—C22—C21	119.0 (4)	C45—C50—H50	119.0
			
C7—P1—C1—C2	−106.3 (4)	C32—P2—C26—C31	−37.0 (4)
C13—P1—C1—C2	17.6 (4)	C38—P2—C26—C31	80.6 (4)
C19—P1—C1—C2	137.8 (3)	C44—P2—C26—C31	−160.0 (3)
C7—P1—C1—C6	67.9 (4)	C32—P2—C26—C27	145.1 (4)
C13—P1—C1—C6	−168.2 (3)	C38—P2—C26—C27	−97.3 (4)
C19—P1—C1—C6	−48.0 (4)	C44—P2—C26—C27	22.1 (4)
C6—C1—C2—C3	0.1 (6)	C31—C26—C27—C28	−0.4 (7)
P1—C1—C2—C3	174.2 (3)	P2—C26—C27—C28	177.5 (3)
C1—C2—C3—C4	0.5 (6)	C26—C27—C28—C29	0.4 (7)
C2—C3—C4—C5	−0.6 (7)	C27—C28—C29—C30	−0.3 (7)
C3—C4—C5—C6	0.0 (7)	C28—C29—C30—C31	0.2 (7)
C4—C5—C6—C1	0.6 (6)	C27—C26—C31—C30	0.3 (7)
C2—C1—C6—C5	−0.7 (6)	P2—C26—C31—C30	−177.7 (4)
P1—C1—C6—C5	−174.9 (3)	C29—C30—C31—C26	−0.1 (7)
C13—P1—C7—C8	−122.5 (3)	C38—P2—C32—C37	152.1 (4)
C1—P1—C7—C8	−0.5 (4)	C26—P2—C32—C37	−89.8 (4)
C19—P1—C7—C8	117.9 (4)	C44—P2—C32—C37	31.9 (4)
C13—P1—C7—C12	60.8 (4)	C38—P2—C32—C33	−33.7 (4)
C1—P1—C7—C12	−177.2 (3)	C26—P2—C32—C33	84.4 (4)
C19—P1—C7—C12	−58.8 (4)	C44—P2—C32—C33	−153.9 (3)
C12—C7—C8—C9	0.6 (6)	C37—C32—C33—C34	0.8 (7)
P1—C7—C8—C9	−176.1 (3)	P2—C32—C33—C34	−173.5 (3)
C7—C8—C9—C10	−0.4 (6)	C32—C33—C34—C35	−1.6 (7)
C8—C9—C10—C11	−0.5 (7)	C33—C34—C35—C36	0.7 (7)
C9—C10—C11—C12	1.2 (7)	C34—C35—C36—C37	0.9 (7)
C10—C11—C12—C7	−1.0 (6)	C35—C36—C37—C32	−1.7 (7)
C8—C7—C12—C11	0.1 (6)	C33—C32—C37—C36	0.8 (7)
P1—C7—C12—C11	176.9 (3)	P2—C32—C37—C36	175.2 (4)
C7—P1—C13—C18	174.7 (3)	C32—P2—C38—C39	−53.1 (4)
C1—P1—C13—C18	53.4 (4)	C26—P2—C38—C39	−171.6 (3)
C19—P1—C13—C18	−67.4 (4)	C44—P2—C38—C39	68.8 (4)
C7—P1—C13—C14	−8.8 (4)	C32—P2—C38—C43	127.4 (4)
C1—P1—C13—C14	−130.1 (3)	C26—P2—C38—C43	8.9 (4)
C19—P1—C13—C14	109.1 (4)	C44—P2—C38—C43	−110.7 (4)
C18—C13—C14—C15	−0.8 (6)	C43—C38—C39—C40	0.3 (6)
P1—C13—C14—C15	−177.3 (3)	P2—C38—C39—C40	−179.2 (3)
C13—C14—C15—C16	0.7 (7)	C38—C39—C40—C41	−0.4 (7)
C14—C15—C16—C17	0.1 (7)	C39—C40—C41—C42	0.2 (7)
C15—C16—C17—C18	−0.9 (7)	C40—C41—C42—C43	0.2 (7)
C16—C17—C18—C13	0.9 (6)	C41—C42—C43—C38	−0.4 (7)
C14—C13—C18—C17	−0.1 (6)	C39—C38—C43—C42	0.1 (6)
P1—C13—C18—C17	176.5 (3)	P2—C38—C43—C42	179.6 (3)
C7—P1—C19—C20	179.6 (3)	C32—P2—C44—C45	73.3 (4)
C13—P1—C19—C20	57.6 (4)	C38—P2—C44—C45	−46.2 (4)
C1—P1—C19—C20	−63.1 (4)	C26—P2—C44—C45	−165.2 (3)
P1—C19—C20—C25	−97.7 (4)	P2—C44—C45—C50	97.8 (4)
P1—C19—C20—C21	83.5 (5)	P2—C44—C45—C46	−82.3 (4)
C25—C20—C21—C22	1.3 (6)	C50—C45—C46—C47	−0.2 (6)
C19—C20—C21—C22	−179.8 (4)	C44—C45—C46—C47	180.0 (4)
C20—C21—C22—C23	−1.3 (7)	C45—C46—C47—C48	0.0 (6)
C21—C22—C23—C24	0.3 (7)	C46—C47—C48—C49	0.7 (7)
C21—C22—C23—Cl5	−178.7 (3)	C46—C47—C48—Cl6	179.7 (3)
C22—C23—C24—C25	0.7 (7)	C47—C48—C49—C50	−1.1 (7)
Cl5—C23—C24—C25	179.7 (3)	Cl6—C48—C49—C50	179.8 (3)
C23—C24—C25—C20	−0.7 (6)	C48—C49—C50—C45	0.9 (7)
C21—C20—C25—C24	−0.3 (6)	C46—C45—C50—C49	−0.3 (6)
C19—C20—C25—C24	−179.2 (4)	C44—C45—C50—C49	179.6 (4)

### Hydrogen-bond geometry (Å, °)

**Table:**

*D*—H···*A*	*D*—H	H···*A*	*D*···*A*	*D*—H···*A*
O1w—H1w1···Cl1	0.84	2.59	3.361 (4)	154
O1w—H1w2···Cl2	0.84	2.41	3.184 (4)	153
O2w—H2w1···Cl1	0.84	2.58	3.385 (9)	161
O3w—H3w1···O4w	0.84	2.31	3.12 (2)	160
O3w—H3w2···Cl3	0.84	2.43	3.27 (2)	172
O4w—H4w1···Cl1	0.84	2.29	3.09 (1)	158
